# Early Extrusion of an Anterior Odontoid Screw: A Case Report

**DOI:** 10.7759/cureus.61915

**Published:** 2024-06-07

**Authors:** Herika Negri Brito, Marcelo Porto Sousa, Lucca B Palavani, Jamal McClendon

**Affiliations:** 1 Neurosurgery, Mayo Clinic, Phoenix, USA; 2 Neurosurgery, Faculty of Medicine, Federal University of Rio de Janeiro, Rio de Janeiro, BRA; 3 Neurological Surgery, Faculty of Medicine, Max Planck University Center, Indaiatuba, BRA

**Keywords:** screw extrusion, odontoid fracture, elderly, dens non-union, atlanto-axial instability

## Abstract

Odontoid fractures in the elderly typically require surgical intervention due to poor adaptability to conservative treatment. Anterior screw fixation, despite its high fusion rates under specific conditions, may lead to complications such as screw extrusion, as demonstrated in the case discussed, necessitating subsequent posterior cervical fusion. This study aimed to describe early extrusion of an anterior odontoid screw and the importance of caution and thorough postoperative assessment in elderly patients undergoing anterior screw fixation for odontoid fracture.

A 73-year-old female patient with a history of ground-level fall and subsequent cervical pain was diagnosed with an odontoid type II fracture and underwent odontoid screw placement in June 2023. However, in August, follow-up imaging revealed screw displacement and a fracture of the posterior arch of the C1 vertebral, which was initially overlooked. After seeking a second opinion, a new surgical approach was decided, involving removal and replacement of the odontoid screw, posterior and posterolateral C1-C2 spinal instrumentation, arthrodesis, and fusion with the use of morselized allograft. The patient was discharged on postoperative day 3 with mild cervical pain, wearing a soft collar, and neurologically intact.

Given the current literature, odontoid screw extrusion rates are still small but can come with enormous potential complications. Also, the present case is a reminder to always double-check preoperative imaging and recognize early failure/malpositioning of hardware.

## Introduction

Odontoid fractures are prevalent in the elderly population. It comprises approximately 18-20% of all cervical injuries and 65-74% of odontoid type II fractures [1]. Management of this type of fracture is either through surgery or conservative halo-vest immobilization. Usually, in the elderly population, the first choice is to keep the patient off the halo-vest and attempt a surgical correction, especially for acute cases, mainly because of the bad adaptive process and high rates of non-union of fracture fragments given the disruption of vascular supply [2].

Surgery is carried out to reduce the disrupted fragments and stabilize the atlantoaxial junction and can be performed through posterior instrumentation and fusion of C1 and C2 vertebrae. Specifically for odontoid fractures, Bohler presented the anterior screw fixation technique in 1982, which only involves the C2 vertebrae and gives the merit of maintaining rotatory motion and cervical spine flexion and extension [3].

Anterior screw fixation offers excellent fusion rates ranging from 89% to 100% if fracture angulation is suitable, the fracture is no older than six months, the patient has good bone quality, and there is an absence of ligament instability or presence of “the odontoid” [4-7].

The aim of this paper is to describe the case of a patient who underwent the removal and replacement of an odontoid screw, followed by posterior cervical fusion, after the early and spontaneous extrusion of the screw due to trauma. Additionally, this case underscores the importance of thoroughly reviewing preoperative imaging and promptly identifying screw failure or misalignment. The patient provided informed consent for the documentation of their case, and this report complies with the guidelines set forth in the CAse REport (CARE) guidelines [8].

## Case presentation

**Figure 1:**
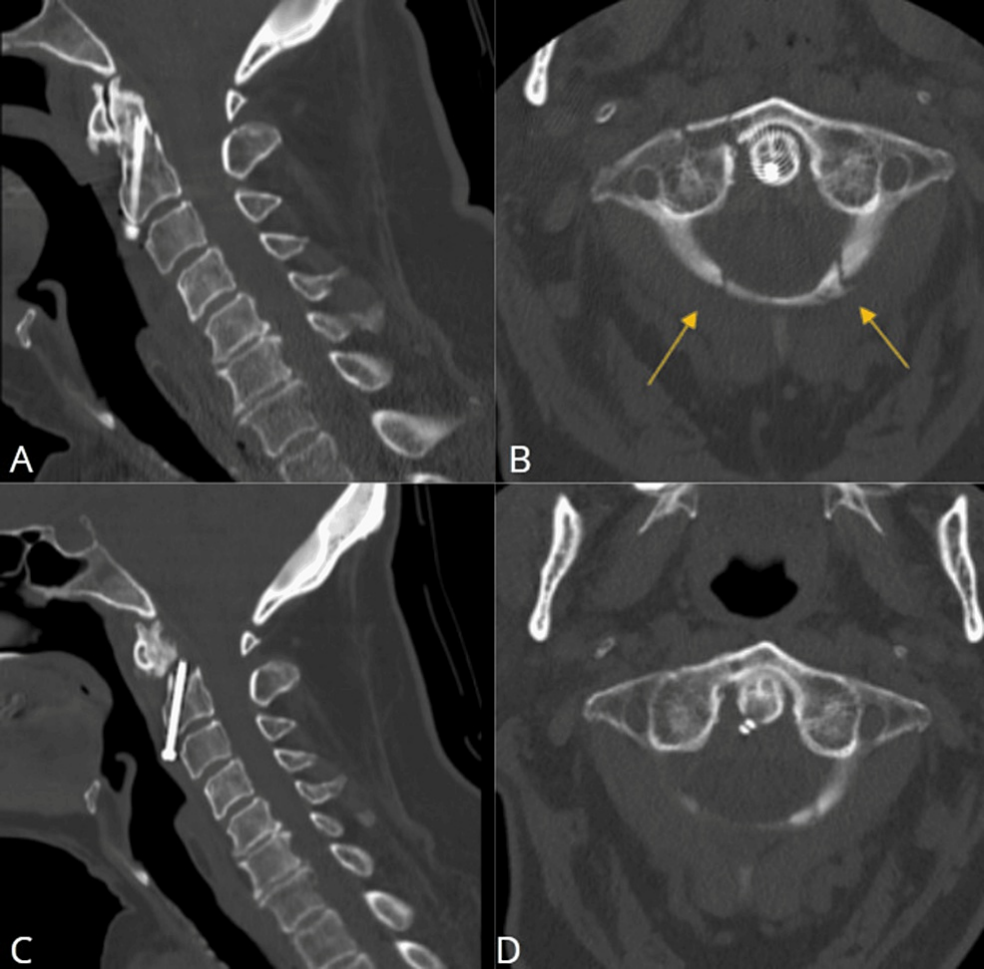
Cervical CT Good positioning of the anterior odontoid screw in sagittal view (A). Axial view (B) shows the tip of the screw and two fracture lines across the posterior arch of C1 (yellow arrows) not previously reported. Cervical sagittal CT demonstrated a single intact screw traversing the C2 vertebral body; however, the screw no longer contacted the dens (C). Another view in the axial image (D).

**Figure 2:**
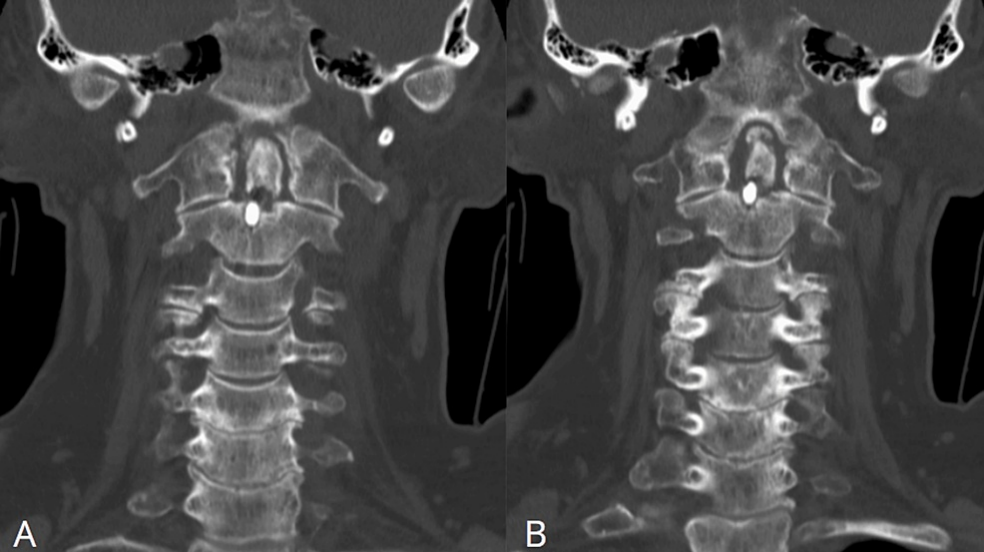
Cervical coronal CT Final positioning of the anterior odontoid screw (A). Good positioning of the anterior odontoid screw (B).

A 73-year-old female patient with a history of osteopenia experienced a recent fall at home, resulting in cervical pain. During hospital admission, an odontoid type II fracture was identified, and the patient was planned for the placement of an odontoid screw in June of 2023 at an outside Institution. She underwent a cervical CT three days after surgery, which showed good screw positioning and reduction of fracture (Figures 1A, 1B, 2A; date: not related).

Later in August, the patient had a follow-up appointment with repeat spine CT (Figures 1C, 1D, 2B; date: August 30, 2023), which showed a single intact screw traversing the C2 vertebral body; however, the screw no longer contacted the dens. The dens were displaced anteriorly and superiorly by approximately 4 mm. Mild periarticular lucency at the distal screw was also observed; otherwise, the craniocervical alignment was anatomic. Upon these findings, maintenance of rigid collar and follow-up imaging had been suggested.

The patient decided to take a second opinion and was seen in our outpatient clinic in September. At the time of the appointment, she had mild cervical pain and had been wearing the rigid cervical collar since the previous surgery with an intact neurological examination. After reviewing her imaging, postoperative axial CT also demonstrated a fracture of the posterior arch of the C1 vertebral (Figure 1B, 2B), not described by the radiologists at the outside institution. Postoperative diagnosis of type II odontoid fracture, atlantoaxial instability, and pseudoarthrosis were made with the decision for a surgical approach.

She underwent removal of the old odontoid screw and replacement with a new one, 40 mm replaced with 46 mm, through the previous anterior cervical approach, followed by posterior and posterolateral approach, C1-C2 spinal instrumentation, arthrodesis, fusion with the use of morselized allograft, and placement of Atlas cable.

**Figure 3:**
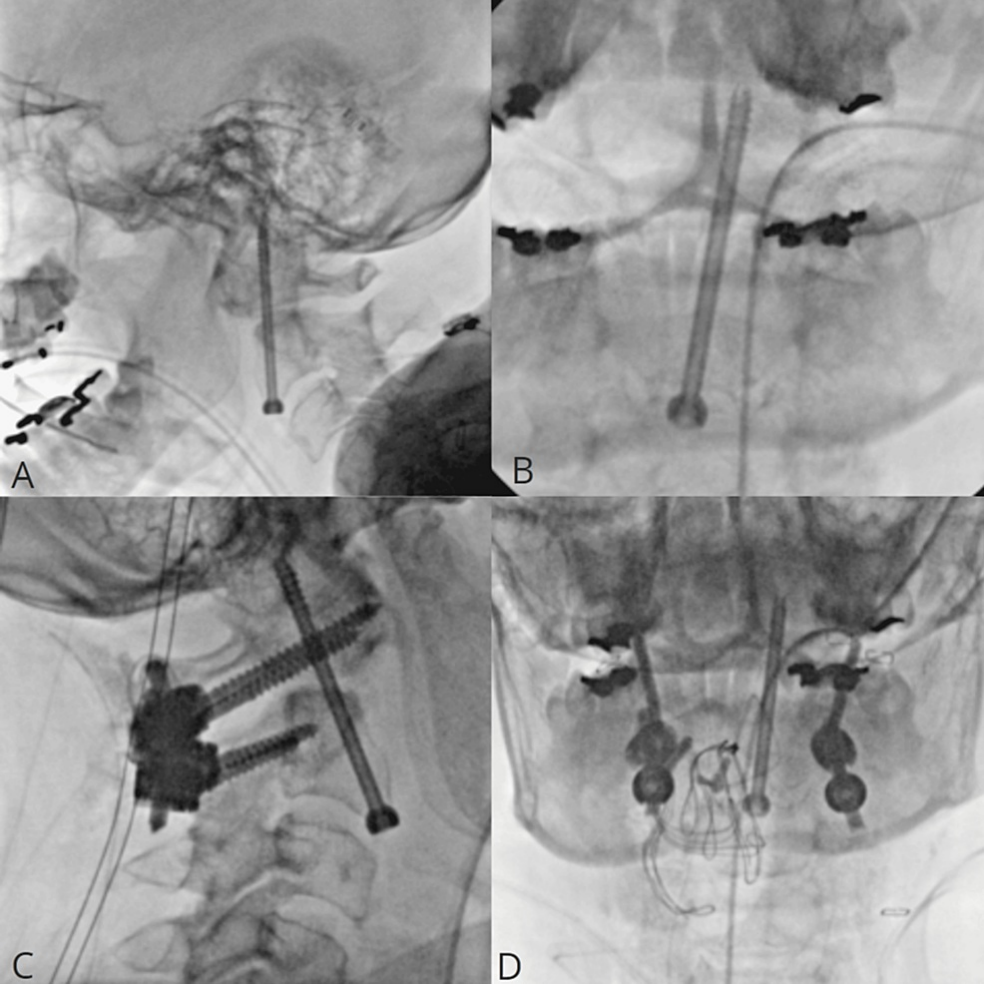
Intraoperative fluoroscopy The final positioning of the anterior odontoid screw in lateral (A) and anteroposterior (B) views. Intraoperative final fluoroscopy demonstrates the positioning of the anterior odontoid screw and screws in C1 pedicle and C2 lateral mass, rods, and Atlas cable in lateral (C) and anteroposterior (D) views.

The patient had satisfactory intraoperative fluoroimaging, showing a reduction of displaced odontoid tip (Figures 3A, 3B: date: September 11, 2023) and good positioning of posterior screws and rods conserving cervical lordosis (Figures 3C, 3D; date: September 11, 2023). She was discharged on postoperative day 3 with mild cervical pain, wearing a soft collar, and neurologically intact.

## Discussion

According to the United States' latest census in 2020, the older population has increased from 4.9 million to 50.9 million since 1920, with a growth rate of approximately 1,000% [9]. Any type of fracture, in this particular population, may represent a challenge for many reasons, and the quality of the bone is one of them.

This patient, in particular, had previous imaging reports that failed to correctly classify the type of odontoid fracture and also to recognize fracture traces in the posterior arch of C1, which could be an early sign of potential instability and the need for additional posterior fusion.

This case is the first in the literature to report early extrusion of the anterior odontoid screw; repair was done through the same previous approach with successful reduction of the anteriorly displaced odontoid tip. Previous studies have reported difficulties in achieving total reduction through the anterior cervical approach. However, a case report has documented successful reduction using the transoral approach, where a hand retractor was used to push the dislocated bone [10]. Several transoral manipulation techniques have also been described in the literature to achieve reduction [11-13].

Subach et al. [7] in their review of anterior odontoid screw fixation found a variety of complications related to instrumentation. From the 252 patients in the series, he found a combined screw malposition rate of 3.2%, a screw pull-out rate of 3.2%, and a screw fracture rate of 0.3%. Previous literature reported that displacements occurred right after surgery or before complete bony fusion, with no pharyngeal perforation [1,14]. In the present case, screw extrusion could be related to combined factors that include not using the adequate size of the screw and failure to recognize that the patient also needed posterior arthrodesis.

Another possible complication connected to the anterior odontoid fixation is esophageal perforation. The overall risk is estimated to be around 1% [15,16]. Lee et al. [17] reported a case of a 27-year-old woman patient who presented to the local ear, nose, and throat clinic three years after anterior odontoid screw placement for type II fracture, with a two-month history of dysphagia and sense of a foreign body in the throat. Laryngoscopy identified the head of the odontoid screw, and radiography showed that it had migrated into the pharyngeal soft tissue.

Other authors also reported a case of esophageal perforation 10 weeks after anterior odontoid screw fixation [18]. The patient in this case had no complaints and no signs of fistula in laryngoscopy, while cervical magnetic resonance imaging demonstrated diffuse inflammation and fistula signs. Fortunately, the patient described in this case report did not have this complication but this could have happened.

## Conclusions

Accurate classification of fractures, particularly in the elderly, is crucial to guide treatment decisions and prevent misdiagnosis of instability, such as C1 posterior arch fractures. Furthermore, postoperative complications associated with anterior odontoid screw fixation, including malpositioning, detachment, and esophageal perforation, require close monitoring. Lastly, early detection of symptoms such as dysphagia is essential for promptly identifying complications, while regular monitoring and imaging examinations are crucial for promptly identifying and resolving problems and optimizing patient care.
